# The Entrainment Frequency of Cardiolocomotor Synchronization in Long-Distance Race Emerges Spontaneously at the Step Frequency

**DOI:** 10.3389/fphys.2020.583030

**Published:** 2021-02-04

**Authors:** Alberito R. de Carvalho, Renan dos S. Coimbra, Eric M. Thomas, Martín C. Rodríguez Paz, Barbara Pellegrini, Leonardo A. Peyré-Tartaruga

**Affiliations:** ^1^Exercise Research Laboratory, Rio Grande do Sul Federal University, Porto Alegre, Brazil; ^2^Integrative Biodynamics Evaluation Laboratory, Western Parana State University, Cascavel, Brazil; ^3^Instituto Politécnico San Arnoldo Janssen, Posadas, Argentina; ^4^Department of Neurosciences, Biomedicine and Movement Sciences, Università Degli Studi di Verona, Rovereto, Italy

**Keywords:** applied kinesiology, biomechanical phenomena, electromyography, locomotion, physiological phenomena, sports

## Abstract

In forced conditions, where the heart rate and step frequency have been matched, cardiolocomotor synchronization (CLS) has been recognized. However, knowledge about the occurrence of CLS and its triggers in sports gesture in real contexts is little known. To address this gap, the current study tested the hypothesis that CLS in running spontaneous conditions would emerge at entrainment bands of muscle activation frequencies associated with a freely chosen step frequency. Sixteen male long-distance runners undertook treadmill assessments running ten three-minute bouts at different speeds (7, 7.5, 8, 9, 10, 11, 12, 13, 14, and 15 km⋅h^–1^). Electrocardiography and surface electromyography were recorded simultaneously. The center frequency was the mean of the frequency spectrum obtained by wavelet decomposition, while CLS magnitude was determined by the wavelet coherence coefficient (WCC) between the electrocardiography and center frequency signals. The strength of CLS affected the entrainment frequencies between cardiac and muscle systems, and for WCC values greater than 0.8, the point from which we consider the emerging CLS, the entrainment frequency was between 2.7 and 2.8 Hz. The CLS emerged at faster speeds (13–15 km⋅h^–1^) most prevalently but did not affect the muscle activation bands. Spontaneous CLS occurred at faster speeds predominantly, and the entrainment frequencies matched the locomotor task, with the entrainment bands of frequencies emerging around the step frequencies (2.7–2.8 Hz). These findings are compatible with the concept that interventions that determine *optima* conditions of CLS may potentiate the benefits of the cardiac and muscle systems synchronized in distance runners.

## Introduction

The muscles adjust their mechanical output to meet the exercise demand. The muscles adjust their mechanical output to meet the exercise demand. The mechanical and metabolic systems interact to face this demand. We defined mechanical systems as segments interconnected by joints producing forces and moments (Roach and Mathalon, 2008). These integrative responses lead to general homeostasis. The nervous system plays a critical role in controlling and tuning muscle function to optimize performance (Neptune et al., 2009). Although motor function as a whole is a consequence of how the body integrates mechanical and metabolic demands, a challenge to understanding the metabolic demand on muscle activation is related to some signals of global metabolic energy expenditure not being identified in the central nervous system. One speculation is that the afferent information sent from the muscle to the central nervous system is responsible for controlling cardiorespiratory response and metabolic energy expenditure (Sparrow, 2000).

According to a paper in which researchers injected microspheres in Guinea fowl blood circulation to scrutinize blood flow distribution at different walking and running speeds, the blood flow of active muscles is regulated aiming to meet the muscle oxygen consumption rate. Approximately 90% of the increase in cardiac output due to exercise was targeted to supply blood flow to active muscles involved with positive work production, and 10% providing increased flow to the coronary circulation and respiratory muscles. Yet, the correlation between blood flow with metabolic energy cost seems valid only for muscular activities that use aerobic routes to obtain energy (Ellerby et al., 2005). In addition, reinforcing the integrative character of the physiological function, there is simultaneous entrainment between some carotid baroreflex end-organ components, such as arterial blood pressure, muscle sympathetic nerve activity, and R–R interval, whose impact on peripheral hemodynamic control measurements ranged from cardiac chronotropic effects to alterations at the level of the skeletal muscle microcirculation. However, the exercise causes some perturbations in this integrative regulation (Wray et al., 2004).

During exercise, the muscle force is adjusted to the mechanical task by the central nervous system, which combines motor unit activation number and frequency as motor unit synchronization. Thus, motor unit recruitment depends on activated fiber type and number (Edstrom and Kugelberg, 1968). Moreover, the histological muscular fiber characteristics, biomechanical properties, and task specificity determine the values of spectral frequency (Bilodeau et al., 2003; Croce et al., 2014). The muscle activity can be measured by exploring the spectral frequency analysis of surface electromyography (SEMG). This method enables estimation of muscle fiber activation and motor unit recruitment as a series of action potentials firing at specific frequencies (Croce et al., 2014).

The effect of muscle contraction on heart rate during cyclic activity investigated under the light of two correlated phenomena, synchronization and entrainment, could bring new rational insights about mechanical and metabolic system interactions in long-distance running, since an optimization of oxygen delivery to the tissue is expected with an impact on metabolic economy and performance in the presence of synchronization (Niizeki, 2005). Synchronization is a phenomenon that encompasses the interaction between at least two independent oscillator systems, and it represents the capacity of systems with different oscillatory frequencies to produce a coincident oscillatory regime due to entrainment between two or more oscillators (Nomura et al., 2001; Bailón et al., 2013). When two or more systems are synchronized, they share common oscillator rhythm inputs; for physiological systems in particular, this is a determinant to ensure the essential rhythms of life (Glass, 2001).

One case of synchronization is the interaction between the cardiac and locomotor systems, so-called cardiolocomotor synchronization (CLS). Locomotor tasks such as walking and running are characterized as cyclic activities that enable the entrainment of heart rate (HR) to the same frequency of step frequency (SF). Hence, the pairing of the HR and SF seems crucial to generate the CLS phenomenon (Kirby et al., 1989; Niizeki et al., 1993; Cerqueira et al., 2017), while entrainment represents the frequencies at which the synchronization emerges. Although not every synchronization condition is caused by entrainment, since the synchronization could emerge just by chance, previous evidence demonstrated that the entrainment during running caused synchronization (Nomura et al., 2001).

It is suggested that in the occurrence of the CLS phenomenon, the cardiac cycle be programmed temporally, as a function of entrainment, to produce a panorama so that the delivery of oxygenated blood to active muscles occurs at the moment when they are most relaxed during the gait cycle and they display the lowest intramuscular pressure (Niizeki, 2005). It has been believed to be a mechanism involved on performance improvement in conditions where synchronization occurs.

The CLS occurs when the locomotor system, represented by muscle activity, entrains the cardiac system to the same frequency. More specifically, the entrainment occurs by matching the HR with the SF; this pairing is often controlled by a metronome that induces the SF to achieve a given HR (Niizeki et al., 1993; Nomura et al., 2001). Challenges with respect to CLS include understanding how it can manifest spontaneously during gait, what determining factors emerge for it, and what its benefits are (Phillips and Jin, 2013). Moreover, CLS has been poorly investigated in spontaneous running conditions without the influence of external factors as a metronome. The question arises as to whether locomotion mechanical parameters are involved in the cardiolocomotor mechanism and if it could improve cardiometabolic efficiency turning beneficial for endurance exercise performance by making the metabolic cost of the task lower.

The muscle activation is elicited by the neural drive, which is the sum of the spiking activities of motor neurons. External oscillators may influence the neural drive, and probably it will be the reason to the coherence between two signals from different sources (Farina et al., 2014). We speculate that SF is an external locomotor condition able to pair, by neural control, the cardiac and muscle oscillator frequencies (Niizeki et al., 1993; Niizeki and Saitoh, 2014; Materko et al., 2015) and that external condition, in turn, could generate a common frequency inducing synchronization of both signals and which can be recognized as entrainment bands of frequencies.

Although CLS is a recognized physiological phenomenon (Niizeki and Saitoh, 2014; Cerqueira et al., 2017), the relationship between speed running and the onset of spontaneous CLS has not been investigated. Furthermore, no study has compared the effects of CLS emergence on entrainment bands of muscle activation frequency, or which SF spontaneous CLS occurs in distance running. As such, this study aimed to identify whether running at a range of different speeds with self-selected SF can induce the emergence of spontaneous CLS and in which entrainment bands of frequencies the CLS emerges. We hypothesized that CLS could emerge spontaneously as a consequence of the frequency of a locomotor task, with entrainment bands of frequencies emerging around the SF, and the CLS may have a preference for muscles whose function is related to the saving of metabolic energy, such as vastus lateralis (VL) and gastrocnemius medialis (GM) muscles whose primary function of their respective muscle–tendon units is to enhance the storage and recovery of elastic energy during running (Monte et al., 2020). Further, understanding the relationship between cardiolocomotor timing and entrainment bands of muscle activation frequency can assist in training prescription and even on pacing and distance running performance.

## Materials and Methods

### Ethical Approval

The institutional ethics committee approved this cross-sectional study (1.115.465) in accordance with the policy statement regarding the use of human subjects in scientific research. All volunteers received clarification regarding the study aims and procedures before inclusion, and all provided formal consent to participate.

### Participants

The participants were 16 proficient male long-distance runners (age 34.1 ± 9.4 years; body mass, 73.1 ± 6.5 kg; stature, 1.75 ± 0.04 m) with running experience in 10- or 21-km races. They were training not less than three times a week for a minimum weekly distance of 30 km and free of the following conditions: (a) systemic diseases; (b) chronic or acute musculoskeletal injuries; and (c) use of drugs that act on the cardiovascular or autonomic systems.

### Instrumentation and Procedures

Each participant was submitted to two sessions of five submaximal bouts of treadmill running interspersed by at least 48 h.

At the first visit, all anthropometric measures and a detailed screening were completed, the best self-reported 10-km race competition times in the last 9 months were recorded, and instruction and familiarization regarding the Total Quality Recovery scale were provided (Kentta and Hassmen, 1998). The running tests were performed on a treadmill (Model 10200/ATL; Inbramed, Porto Alegre, Brazil) with a fixed incline of 1%. Because the SF on the treadmill may differ from the SF on the ground, the individuals ran for a couple of minutes and could familiarize the running technique to treadmill condition. The runners undertook treadmill assessments on two different days, running in each day five 3-min bouts at distinct speeds (7, 7.5, 8, 9, 10, 11, 12, 13, 14, and 15 km⋅h^–1^). The order of the speeds was randomized over 2 days, except the first speed of each day that was always 7 or 7.5 and defined randomly and utilized as a warm-up. During the last 2 min of running in each bout, the SEMG and electrocardiography signals (ECG) were recorded. The duration between consecutive bouts was determined considering two criteria: (a) the subject felt fully recovered by Total Quality Recovery scale and (b) heart rate decreased below 115% of resting heart rate.

The ECG and SEMG of the *vastus lateralis* and *gastrocnemius medialis* muscles were recorded with the same signal conditioner to determine the center frequency and CLS (NewMiotool with eight channels, 16-bit resolution, and automatic gain; Inbramed, Porto Alegre, Brazil) at a 2,000-Hz sampling frequency in which one biopotential channel was configured to capture ECG (Vanderlei et al., 2008). The skin impedance was reduced by shaving, abrasion, and washing the skin with cotton and alcohol to fix the Ag/AgCl electrodes (10-mm diameter). The muscle electrodes were placed on the right VL and GM muscles according to Surface ElectroMyoGraphy for the Non-Invasive Assessment of Muscles recommendations (Hermens et al., 1999). To collect the ECG, two electrodes were placed on the manubrium sternal and the fifth intercostal space at the point crossing the left midclavicular line (CM5 lead) (Vanderlei et al., 2008). The reference electrode was placed on the right anterior iliac spine.

### Data Processing

#### Frequency Spectrum of Each Signal (SEMG and ECG) and Their Respective Means

First, to decompose the signals in their spectrum of frequencies, we used a Wavelet code from a MATLAB environment (R2015b; Natick, MA, United States), Wavelet Packet Decomposition 1-D (“wpec” function, level 6, and wavelet mother symlet14). Symlet14 is a wavelet mother associated with 28 low-pass decomposition filters and 28 high-pass decomposition filters. The decomposition generated a matrix with 64 columns matching the scales and the rows correspondent to the length of the signal vector. Subsequently, we applied the “wpspectrum” function from MATLAB, which returned a matrix of Wavelet Packet Power spectrum based on the Wavelet Packet Transform. Then, for each column containing the scale with their respective frequencies, we calculated the mean of these frequencies that resulted in a row vector 1 × 64. Finally, we included the frequencies on that row vector in ascending order (from scale 1 to scale 64) and determined the center frequency as the mean of this vector (SEMG_MNF_). We considered the mean value as representative of the frequency spectrum.

From the ECG signal, the HR was calculated from the reciprocal of R–R time in Hertz.

#### Finding the Range of Frequencies of Each Signal

We resorted to signal decomposition to determine the range of frequencies composing each signal to feed the steps necessary to further analyze synchronization and entrainment. The frequencies corresponding to the first scale and the scale 57, representing 90% of the spectrum, and the last scale, 64, were determined (Table 1).

**TABLE 1:** Frequencies found at scales 1, 57, and 64 of the row vectors resulting from the decomposition process for all signals.

	Scale 1 (Hz)	Scale 57 (Hz)	Scale 64 (Hz)
	Mean ± SD	Mean ± SD	Mean ± SD
ECG	6.7 ± 4.3	60.5 ± 25.0	903.1 ± 224.5
SEMG–VL	4.4 ± 2.4	91.8 ± 34.0	824.1 ± 882.5
SEMG-GM	7.4 ± 2.9	198.7 ± 69.1	286.5 ± 104.4

*Scale 57 corresponds to 90% of the signal content. The values represent all speeds and participants. ECG, electrocardiogram signals; SEMG-VL, electromyography of the vastus lateralis; SEMG-GM, electromyography of the gastrocnemius medialis.*

As the magnitude of the frequencies has been high between scales 57 and 64, this interval contained only 10% of the signal content; for data from the present study, we only used the frequencies between scales 1 and 57 to calculate the scale range used in the wavelet coherence analysis. We adopted a frequency range spectrum of 1.5–350 Hz. The conversion from the frequency range of the scale range (Scalerange) was performed according to equation 1:

(1) Scalerange=fc/[freqrange×(1/fs)]

where fc is the central frequency of the wavelet mother, freqrange is the frequency range spectrum, and fs is the sampling frequency. The fc can be calculated by the “centfrq” function from MATLAB. The mother wavelet used was Morlet. The Scalerange was minimum scale (S0) = 4.6429 and maximum scale (MxS) = 1.0e + 03 × 1.0833.

#### Determining Synchronization and Entrainment

We investigated the CLS by determining the interaction between two temporal series and return wavelet coherence coefficient (WCC). We used an adapted code of wavelet transform coherence (WTC) from wavelet coherence. The MATLAB package was presented in the literature (Cui et al., 2012) and is available for free download on the Aslak Grinsted website (Aslak Grinsted Web Site [Internet], 2019). For WTC coding, we used the following settings: Monte Carlo Count = 300, mother wavelet = Morlet, S0, and MxS; the last two settings were determined in the previous step.

The code returned, for each pair of temporal series, electrocardiography and surface electromyography of the *vastus lateralis* (ECG-VL), and electrocardiography and surface electromyography of the *gastrocnemius medialis* (ECG-GM), a matrix with 95 columns matching the WCC and the rows corresponding to the length of the temporal series pair vector. As the goal of the procedure was to determine the WCC values that represent the synchronization during the running test, we calculated the WCC means for each column, which resulted in a row vector 1 × 95 with the corresponding WCC to each scale. From the WTC code, the scale brings the real period information (Cui et al., 2012; Domingues et al., 2016). We obtained in this processing a row vector 1 × 95 with the respective period for each of 95 scales. The conversion from period to frequency, in Hz, was done accordingly:

(2) $${\text{Frequency}}_{\text{hz}}=\left[1/\left(\text{period}/fs\right)\right]$$

where the period is the real period of the code and fs is the sampling frequency.

At the end of processing, the WCC, period, and entrainment frequency were determined for each scale. The WCC is a parameter estimating the degree of linearity of the interaction between two temporal series, and it works as a localized correlation coefficient in the time-frequency scale. The WCC varies from zero (0) to one (1), where one indicates a linear correlation between two functions around time *t* and scale *a*. For a WCC equaling zero, there is no interaction. As the WCC is analogous to the squared Pearson determination *r*^2^, to assess WCC strength, we used the same criteria used for the strength evaluation of the squared Pearson value. The WCC strength magnitude reflects the proportion of variance of the temporal series X at a frequency (f) considered in the constant linear transformation of the complex spectral coefficients derived from temporal series Y (Roach and Mathalon, 2008). The WCC strength magnitudes were very weak (0–0.19), weak (0.20–0.39), moderate (0.40–0.59), strong (0.60–0.79), and very strong (0.8–1) (Jabeen et al., 2016). We assumed that the spontaneous CLS emerged at a WCC ≥ 0.8.

**FIGURE 1:**
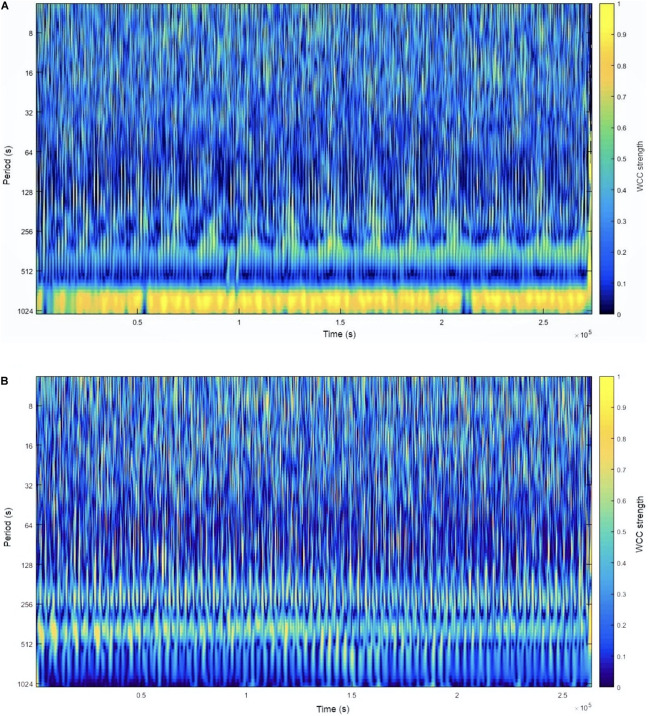
A graphic interpretation of CLS by WCC strength analysis at 14 km⋅h^–1^. Panel **(A)** shows one speed condition where the CLS was weak in almost all real periods. Panel **(B)** shows another speed condition in which there was a band of the real period in which the CLS was very strong at all running test durations. The WCC strength can be observed by WCC in the column on the right of the graph. The yellow color represents a WCC close to 1, while the dark blue color represents a WCC close to 0. CLS, cardiolocomotor synchronization; WCC, wavelet coherence coefficient.

Figure 1 shows some examples of the visual interpretation of CLS by WCC strength analysis.

### Statistical Analysis

For the statistical analysis, we used Generalized Estimating Equations, which is based on the maximum likelihood and which uses Wald’s chi-square test (Wald χ2) to identify the effect of the variable on the generalized linear model, with Bonferroni test as the *post hoc* test.

We tested the goodness of fit for scale response for both model types, linear and gamma with log link, and we chose the model with the smallest value of the quasi-likelihood under the independence model criterion. For all analyses tested, the gamma model had the best fit. First, we count the number of coefficients (events) classified as such for each WCC strength category. Still, we analyzed HR and SF at different speeds considering the SF as the locomotor task frequency. As the speeds were fixed, they were used as a categorical variable. To investigate if the CLS emerged spontaneously, the speed was the factor and all WCC coefficients for each pair of temporal series (ECG-VL and ECG-GM), at each speed, were considered the dependent variable. Finally, we investigated the entrainment frequencies and the center frequency of the VL (SEMG_MNF_-VL) and the center frequency of the GM (SEMG_MNF_-GM) as dependent variables for each WCC strength category.

*A posteriori* power analysis was performed to confirm a minimum power of 0.8 using the software GLIMMPSE (Munjal et al., 2014).

## Results

We conducted a power analysis (*a posteriori*) for SF and WCC outcomes, and both achieved a power higher than 0.8. The main sample categorization variables were weekly training volume (53.4 ± 27.9 km) and mean of the best performance times in the 10-km race (41.4 ± 3.7 min). Taking into consideration the 16 participants, 10 speeds, 95 coefficients, and their respective entrainment frequencies per test, 15,200 events were obtained for a given pair of temporal series. The distribution of these events in absolute and percentage values and strength WCC categories are shown in Table 2.

**TABLE 2:** Number of events observed at different WCC strength categories for each temporal series pair analyzed.

Strength WCC categories	ECG-VL temporal temporal series pair		ECG-GM temporal series pair	
		
	Events	Percentage events	Events	Percentage events
Very weak	5,704	37.5%	5,873	38.6%
Weak	8,690	57.2%	8,277	54.5%
Moderate	565	3.7%	734	4.8%
Strong	216	1.4%	261	1.7%
Very strong	25	0.2%	55	0.4%
TOTAL	15,200	100%	15,200	100%

*ECG, electrocardiogram signals; VL, vastus lateralis; GM, gastrocnemius medialis; WCC, wavelet coherence coefficient.*

**FIGURE 2:**
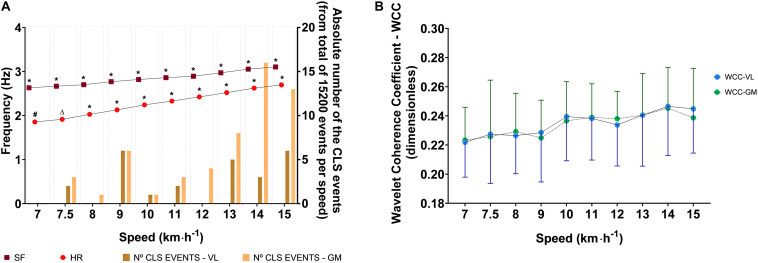
Descriptive (mean and 95% CI) and analytic statistical analysis of effects of running speed on physiological (HR and WCC) and mechanical (SF) variables during running tests. **(A)** Effects of the running speed on the step frequency and heart rate considering the absolute number of events in which the WCC values indicated CLS (in the very strong category) in each speed. **(B)** Effects of running speed on WCC coefficients values for the VL and GM (ECG-VL and ECG-GM). HR, heart rate; SF, step frequency; VL, vastus lateralis; GM, gastrocnemius medialis; WCC, wavelet coherence coefficient; CLS, cardiolocomotor synchronization. In the panel **(A)**, (*) represents a significant difference between speeds; (#) represents significant differences between speeds except 7.5 km⋅h^–1^; (Δ) represents a significant difference between speeds except 7.0 km⋅h^–1^.

The speed affected the HR [Waldχ^2^(9) = 714.792; *p* < 0.001] and the SF [Waldχ^2^(9) = 315.712; p < 0.001]; thus, both increased as speed increased, as can be seen in Figure 2 by descriptive and inferential statistics. However, the speed did not affect the WCC coefficient values for temporal series ECG-VL [Waldχ^2^ (9) = 52.3; *p* > 0.05] and temporal series ECG-GM [Waldχ^2^ (9) = 36.6; *p* > 0.05]. At all speeds, for both temporal series, the WCC coefficients were classified as weak synchronization. The descriptive and inferential statistical analyses are shown in Figure 2.

**FIGURE 3:**
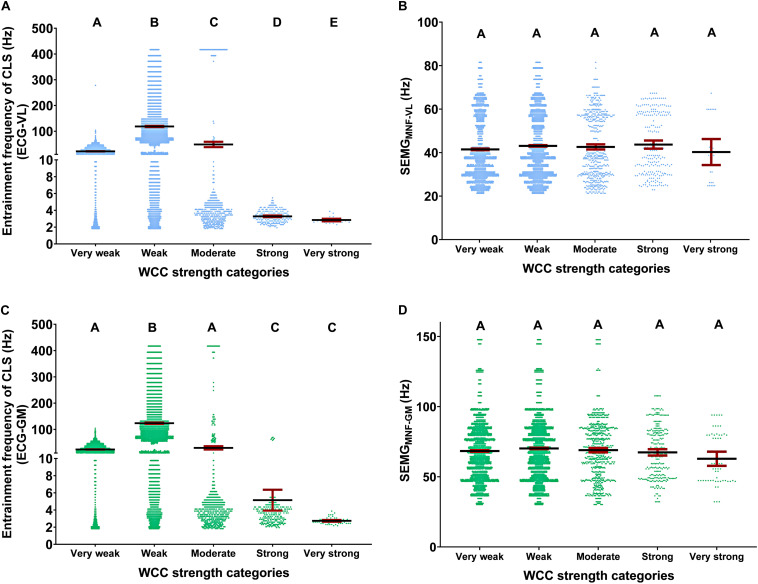
Descriptive (mean and 95% CI) and statistical analysis of the WCC strength categories effects, regarding CLS, during running tests, on center frequency of the muscle spectral density, and at the entrainment frequencies. Descriptive analysis was shown as the unitary distribution of all events around the 95% confidence interval per WCC strength categories. Effects of the WCC strength categories at the entrainment frequencies for ECG-VL **(A)** and ECG-GM **(C)** and on center frequency of the spectral density to vastus lateralis (SEMGMNF-VL) **(B)** and *gastrocnemius medialis* (SEMGMNF-GM) **(D)**. The WCC strength magnitudes were very weak (0–0.19), weak (0.20–0.39), moderate (0.40–0.59), strong (0.60–0.79), and very strong (0.8–1). ECG, electrocardiography; VL, vastus lateralis; GM, *gastrocnemius medialis*; WCC, wavelet coherence coefficient; CLS, cardiolocomotor synchronization. Different letters indicate statistical differences between WCC strength categories (*p* < 0.05). For example, A is different from B, B is different from C, and so on, and equal letters indicate statistical equality.

The WCC strength categories affected the entrainment frequencies for ECG-VL [Waldχ^2^ (4) = 8568.073; *p* < 0.001] and ECG-GM [Waldχ^2^ (4) = 5954.384; *p* < 0.001], although they did not influence the SEMG_MNF_-VL [Waldχ^2^ (4) = 5.492; *p* > 0.05] or the SEMG_MNF_-GM [Waldχ^2^ (4) = 2.807; *p* > 0.05]. The descriptive and inferential statistical analyses are shown in Figure 3.

## Discussion

While preceding studies have examined the role of exercise mode on CLS, the mechanisms responsible remain elusive due to the minimal simultaneous use of neuromuscular and cardiac assessments and due to narrow speeds used. This study investigated whether the CLS emerges spontaneously at a range of running speeds, with a self-selected SF, and in which entrainment frequencies the CLS emerges. A novel aspect of this study was to further elucidate if CLS occurs with changes in running speed, and if these events would change the activation frequency produced by skeletal muscle during these tasks. Our hypothesis that spontaneous CLS could emerge spontaneously was partially confirmed since it occurred, and the CLS was insufficient to affect neuromuscular activation. Importantly, although low in number, the events influenced the entrainment frequency bands and concurred with the locomotor task.

Other studies that investigated cardiolocomotor entrainment during running reported similar findings. Bailón et al. (2013) identified one frequency centered in the stride frequency in the components of frequencies of the HR variability. This frequency centered in the stride frequency was a consequence of cardiolocomotor entrainment, but the authors recognized that it is not easy to identify it because that entrainment frequency was overlapping the autonomic nervous system frequencies (Bailón et al., 2013). In the other study (Materko et al., 2015), just one participant presented short epochs of phase synchronization, but this occurred at the frequency compatible with the sympathetic nervous system band of frequency.

The human skeletal muscles show considerable heterogeneity in their muscle fiber composition, which favors distinct physiological and mechanical attributes. Hence, the recruitment pattern of the motor unity is intricate and the surface myoelectrical signal brings information from all fiber types together. Nonetheless, the size principle is a robust framework that describes the motor unity recruitment pattern with some evidence supporting it (Wakeling and Rozitis, 2004; Hodson-Tole and Wakeling, 2009; Croce et al., 2014). Taking it into account, our hypothesis was that the SEMG would be affected by the speed, and at faster speeds we expected higher electromyographic activity of the muscles with higher depolarization frequency due to the recruitment of larger motor unities in comparison with slower speeds.

However, an increasing body of evidence shows that the size principle is not applied under any conditions, especially as response to the mechanical demand of locomotion (Hodson-Tole and Wakeling, 2009). We speculated about a particular motor unity pool, called task groups, that responds to multiple distinct tasks. Such independent task groups could have different central connections, recruitment patterns, and intrinsic properties optimized for a specific functional task. Still, the mechanical demand of the motor task tends to be selective to the faster motor unity population; when analyzing the motor unity recruitment, the interaction between the muscle and the musculoskeletal system as a whole and not just the fiber type must be considered. The intrinsic features of muscle, like activation–deactivation kinetics and force–velocity properties, seem to affect motor unity recruitment in locomotor tasks (Hodson-Tole and Wakeling, 2008, 2009; Lee et al., 2013; Moore, 2016).

Cavagna et al. (1997) conducted a series of experiments and observed that the lowest metabolic energy expenditure occurs when the body operates at the resonant frequency of the bouncing system. When an oscillatory system (such as the bouncing system) undergoes an external periodic perturbation (for instance, SF) whose frequency equals its natural frequency, this phenomenon is denominated resonance. The resonant frequency at running can be defined as that frequency that requires the lowest metabolic energy amount to maintain the bouncing oscillation. These authors tested several running speeds and observed that the resonant frequency occurred when the self-selected SF and the natural frequency of the spring-mass system were coincident at 2.6–2.8 Hz, which reinforced our hypothesis that the CLS events are a consequence of locomotor tasks, since the entrainment frequency in the very strong category was 2.7–2.8 Hz too, suggesting that the system oscillatory frequency induced by the SF and the natural frequency were coincident, probably to meet the metabolic demand.

A previous study (Monte et al., 2020) reported that the GM muscle fascicle was operating toward smaller lengths at faster running speeds, providing more elastic energy during the propulsion phase. On the other hand, the VL muscle fascicle was operating in longer lengths at faster running speeds without significant changes in elastic loading (negative mechanical power) or recoil (positive mechanical power) performed by series elements. Although the elastic energy recovery was not observed, the VL fascicles had a quasi-isometric behavior during running at increasing speeds, resulting in a reduced “Fenn effect” (extra energy mobilized in shortening muscle contraction in comparison to isometric contraction). Integratively, the muscle–tendon unit of GM lengthened and shortened more and recoiled more elastic energy than the muscle–tendon unit of VL. These findings may explain why the number of CLS events was higher in GM. Moreover, based on that paper (Monte et al., 2020), the speed at which both the VL and GM were the closest to optimal length was 10 km⋅h^–1^, similar to our speed, at 9 km⋅h^–1^, whose CLS was most frequent.

The entrainment frequencies at the very strong category were 2.7–2.8 Hz. When converting those frequencies in Hertz to cycles per minute, the values found are 162 and 168 cycles⋅min^–1^. The metabolic cost of running shows a U-shaped curve with SF, and the optimal SF corresponding to the lowest metabolic cost is approximately 170 steps per minute (de Ruiter et al., 2014). It has been suggested that experienced runners tend to choose their self-selected SF close to the optimal SF. Consequently, runners select an optimal SF due to energetic requirements. Although the presence of CLS might be a mode of mechanistically linking mechanical and physiological processes that improve locomotor performance, the correlations between CLS and metabolic repercussions on performance have not yet been explored. At a given speed, there is a wide range of biomechanical and physiological responses arising from aerobically trained individuals (Moore, 2016). Perhaps including the CLS as a function of fixed speed might overshadow the occurrence of CLS. Instead, in future studies, we suggest looking for neuromuscular, biomechanical, and physiological changes in running under specific speeds at which CLS occur.

The primary finding of Constantini and colleagues’ research was that synchronizing foot strike with the diastolic phase of the cardiac cycle in submaximal speeds running is able to reduce HR and induce positive metabolic effects in elite distance runners once provides a cardiac advantage for better performance. They suggested that if diastolic and locomotor phase timings are synchronized, it could cause an increase in stroke volume and/or possibly enhanced coronary and skeletal muscle perfusion (Constantini et al., 2018).

It is worth noting that the CLS events, especially in the GM, occurred most frequently at faster speeds at which the SF and HR were closer. In the present study, the differences between SF and HR were higher than expected for CLS conditions. These differences in CLS are around 1% (Kirby et al., 1989; Cerqueira et al., 2017). Therefore, the sparse occurrence of CLS was probably due to distinctive values of SF and HR. Future studies in this field should include combinations of speed and incline capable of approaching the SF and HR.

There is likely a limited range of exercise intensities in which the oscillatory rhythms of both systems, cardiac and locomotor, are spontaneously synchronized. These intensities correspond to those in which the need to bring blood flow to supply the active muscles or the cardiac load to attend to the metabolic demand are close to their limits (Kirby et al., 1989). According to findings of the present study, the SF was higher than the HR and we speculate that the spontaneous CLS events may emerge at high speeds or after long-term endurance running when the cardiovascular system is close to the maximum overload.

Acute interventions on SF or gesture technique may alter running mechanics and energetics in distance runners, particularly with potential effect in the loading rate. Some studies have repeatedly shown that different interventions, including SF and shoes (Warne et al., 2017), reduce impact forces and alter the running economy. Our study represents a start point for these interventions to better understand the mechanisms evolved on these mechanical and energetic alterations. The CLS seems to be a candidate mechanism to mediate these alterations affecting, possibly, the mechanical efficiency (Roach and Mathalon, 2008).

Some limitations of the present study should be pointed out. A possible difference between the ground and the treadmill running mechanics may have influenced the results. However, this difference is known, as cited in the Methods section. Our runners had only intermediary training volume; therefore, our findings may not be generalized to runners with higher training load. Another limitation was the sample composed only of men in an attempt to avoid the effects of the female hormone cycle, but which, on the other hand, prevents the generalization of findings.

In conclusion, the spontaneous CLS occurred inside the range of speeds tested (7.0–15 km h^–1^), but the occurrence of CLS did not change the activation frequency in skeletal muscle. Therefore, the entrainment frequencies matched the locomotor task, with the entrainment bands of frequencies emerging around the SF (2.7–2.8 Hz). These findings are compatible with the concept that interventions that determine the optimal conditions of CLS may potentiate the benefits of the cardiac and muscle systems synchronized in distance runners.

## Data Availability Statement

The original contributions presented in the study are included in the article/supplementary material, further inquiries can be directed to the corresponding author/s.

## Ethics Statement

The studies involving human participants were reviewed and approved by Universidade Federal de Ciências da Saúde de Porto Alegre. The patients/participants provided their written informed consent to participate in this study.

## Author Contributions

This study was developed in the Exercise Research Laboratory, Rio Grande do Sul Federal University. AC, BP, and LP-T took part in the conception of the study. AC, RC, and ET collected and analyzed the data. MP, BP, and LP-T contributed to conduct of the experiments and data analysis. AC and LP-T drafted the manuscript. All authors contributed to revision of the manuscript. All authors approved the final version of the manuscript and agreed to be accountable for all aspects of the work in ensuring that questions related to the accuracy or integrity of any part of the work are appropriately investigated and resolved. All persons designated as authors qualify for authorship, and all those who qualify for authorship are listed.

## Conflict of Interest

The authors declare that the research was conducted in the absence of any commercial or financial relationships that could be construed as a potential conflict of interest.
